# Intracranial Injection of Dengue Virus Induces Interferon Stimulated Genes and CD8^+^ T Cell Infiltration by Sphingosine Kinase 1 Independent Pathways

**DOI:** 10.1371/journal.pone.0169814

**Published:** 2017-01-17

**Authors:** Wisam H. Al-Shujairi, Jennifer N. Clarke, Lorena T. Davies, Mohammed Alsharifi, Stuart M. Pitson, Jillian M. Carr

**Affiliations:** 1 Microbiology and Infectious Diseases, School of Medicine, Flinders University, Adelaide, South Australia, Australia; 2 Centre for Cancer Biology, University of South Australia and SA Pathology, Adelaide, South Australia, Australia; 3 Vaccine Research Laboratory, Research Centre for Infectious Diseases, and Department of Molecular and Cellular Biology, School of Biological Sciences, University of Adelaide, Adelaide, South Australia, Australia; Institut Pasteur of Shanghai Chinese Academy of Sciences, CHINA

## Abstract

We have previously reported that the absence of sphingosine kinase 1 (SK1) affects both dengue virus (DENV) infection and innate immune responses *in vitro*. Here we aimed to define SK1-dependancy of DENV-induced disease and the associated innate responses *in vivo*. The lack of a reliable mouse model with a fully competent interferon response for DENV infection is a challenge, and here we use an experimental model of DENV infection in the brain of immunocompetent mice. Intracranial injection of DENV-2 into C57BL/6 mice induced body weight loss and neurological symptoms which was associated with a high level of DENV RNA in the brain. Body weight loss and DENV RNA level tended to be greater in SK1^-/-^ compared with wildtype (WT) mice. Brain infection with DENV-2 is associated with the induction of interferon-β (IFN-β) and IFN-stimulated gene (ISG) expression including viperin, Ifi27l2a, IRF7, and CXCL10 without any significant differences between WT and SK1^-/-^ mice. The SK2 and sphingosine-1-phosphate (S1P) levels in the brain were unchanged by DENV infection or the lack of SK1. Histological analysis demonstrated the presence of a cellular infiltrate in DENV-infected brain with a significant increase in mRNA for CD8 but not CD4 suggesting this infiltrate is likely CD8^+^ but not CD4^+^ T-lymphocytes. This increase in T-cell infiltration was not affected by the lack of SK1. Overall, DENV-infection in the brain induces IFN and T-cell responses but does not influence the SK/S1P axis. In contrast to our observations *in vitro*, SK1 has no major influence on these responses following DENV-infection in the mouse brain.

## Introduction

Sphingolipids, which are an integral part of membranes in all eukaryotic cells, have been involved in a variety of cell signalling functions [1]. One of these signalling sphingolipids is sphingosine-1-phosphate (S1P) which has well-known critical roles in cell growth and development [2] and T-lymphocyte recruitment [3]. This latter property has drawn considerable recent attention and has led to the clinical use of S1P analogues in the treatment of diseases such as multiple sclerosis [4]. S1P is a normal metabolite produced following the phosphorylation of sphingosine by the sphingosine kinases (SKs) which can play an important role in cell signalling and inflammation [5]. For example, a recent study by Harikumar *et al*., showed that SK1 was required for interferon regulatory factor (IRF)-1- mediated induction of CXCL10 and CCL5 chemokines following interleukin 1 (IL-1) stimulation [6]. There are two forms of SK, designated SK1 and SK2 [7]. Both isozymes show a high degree of sequence similarity although they vary in size, catalytic properties, tissue distribution, and subcellular localisation [8], and have been proposed to have complementing but also distinct physiological roles [1, 9].

A role for SK1 during viral infections is emerging, as we have recently reviewed [10]. Several viruses have been shown to modulate the activity or level of SK1 for efficient viral infection. For instance, bovine viral diarrhoea virus (BVDV) reduced the catalytic activity of SK1 to promote viral replication [11]. In contrast, an increase of SK1 expression and activity during human cytomegalovirus (HCMV) [12], influenza A virus [13], and measles virus (MV) [14] infections enhanced viral replication. Moreover, SK1 can promote viral infections through different biological mechanisms. SK1 has been shown to prolong survival of virus-infected cells [12], and viral protein synthesis [14]. We have previously shown that SK1 activity is altered during dengue virus (DENV) infection [15, 16], and that a reduction in SK1 affects the ability of DENV to induce interferon-stimulated genes (ISGs) *in vitro* [17]. Here, we investigated the effect of SK1 on DENV replication and the induction of ISGs using an *in vivo* model comprising intracranial (ic) injection of DENV.

DENV is a globally important human pathogen that can cause a wide spectrum of clinical presentations from a febrile illness to a life threatening infection with bleeding complications [18]. The disease severity and pathogenicity is thought to be immune-mediated in which immune responses to DENV exacerbate damage to the host [19–21]. Unlike DENV-infection in humans, DENV does not replicate well or cause symptoms reflective of human disease in immunocompetent wild type (WT) mice [22], but can cause a similar pathology in mice deficient in the interferon (IFN) response, such as in the AG129 IFN receptor knockout mouse model [23]. Analysis of neurovirulence—the induction of symptoms of brain infection including reduction in hind limb function has been a widely used historical method to indicate the presence of virus following ic inoculation. Ic injection of DENV into WT mice, although not reflective of a natural mode of DENV-infection, is associated with DENV replication and neurological symptoms [24, 25] but additionally, may reflect some aspects of DENV-associated neurological disease in humans [26]. Although DENV does not antagonise IFN responses in mice as it does in humans [27], in our study we utilised the DENV ic mouse infection model as a means to assess the role of SK1 in DENV infection and induction of ISGs *in vivo* in an immunocompetent animal. We compared virus replication and immune responses following ic injection of DENV into WT and SK1^-/-^ mice. Our data define novel ISG and T-cell responses, and a lack of change in the SK/S1P axis in the brain following DENV infection and demonstrate that SK1 is not a key regulator of these processes in the brain.

## Materials and Methods

### Ethics statement

All animal procedures were performed in accordance with Flinders University Animal Welfare committee approval number 870/14 and Institutional Biosafety Committee approval NLRD 2011–10.

### Mice

Three to four weeks of age WT C57BL/6 (n = 24) and homozygous knockout for the *Sphk1* gene encoding SK1 (SK1^-/-^) (n = 17) [28] mice were used in this study. All mice were kept in a pathogen-free environment on a 12 hours cycle of light and darkness with *ad libitum* access to food and water.

### Virus production

Mice were infected using MON601, a full-length cDNA clone of DENV-2 New Guinea C strain [29]. The virus stock was produced from *in vitro* transcribed RNA that was transfected into baby hamster kidney clone 21 (BHK-21) cells and amplified in *Aedes albopictus* C6/36 cells. Cell culture supernatants containing virus was harvested, clarified, filtered, and stored at– 80°C. The titre of infectious virus was determined by plaque assay using African green monkey kidney (Vero) cells and quantitated as plaque forming unit per ml (pfu/ml).

### DENV-2 challenge and follow-up

WT and SK1^-/-^ mice were anaesthetised by isoflurane inhalation, and infected by ic injection with 800 pfu of DENV-2 MON601 diluted in phosphate-buffered saline (PBS) in a volume of 10 μl. Mock control mice were injected ic with PBS. Animals were visually monitored twice daily for signs of DENV-induced neurological disease including slow movement, hunched posture, or reduction in hind limb function. In addition, animals were monitored for body weight. Any sign of neurological disease or loss of more than 10% of body weight represented a termination point and animals were sacrificed immediately by isoflurane anaesthetic inhalation and humane decapitation. Brain tissues were harvested at sacrifice and divided into two parts. The ipsilateral section was resuspended in TRIzol reagent (Ambion Life Technologies) for RNA extraction and real-time quantitative PCR (qRT-PCR) analysis and the contralateral section was snap frozen in liquid nitrogen for SK activity assay and S1P quantification or fixed for histological analysis.

### Real-time quantitative RT-PCR

Total RNA was extracted from brain tissues using TRIzol (Ambion Life Technologies), according to the manufacturer’s instructions. The extracted RNA was DNase I treated (Zymo Research) and quantitated by spectrophotometry (NanoDrop elite, Thermo Scientific). Total RNA (0.5 μg) was reverse transcribed using M-MuLV reverse transcriptase (NEB) and random hexamers (NEB) in a 20 μl final volume, and subjected to a real-time qRT-PCR. qRT-PCR was carried out using 2 μl of cDNA template in a 10 μl reaction using iTaq SYBER green (BioRad) with 20 μM of each forward and reverse primer. All PCR primers were synthesised by GeneWorks with sequences as listed in Table 1. Real-time qRT-PCR was performed using Rotor-Gene 3000 real-time PCR system (Corbett research, Australia) under the following conditions: one cycle of 95°C for 5 minutes; 45 cycles of 95°C for 15 seconds, 59°C for 30 seconds, and 72°C for 30 seconds; and one cycle of 72°C for 60 seconds followed by melt curve analysis. Quantitative DENV copy number was calculated from a standard curve generated from known concentration of MON601 DNA quantitated by spectrophotometry. DNA copy numbers from 15 pg/μl to 0.015 pg/μl were analysed in concurrent real-time PCR to generate a standard curve from which unknown DENV RNA copy numbers were calculated. Relative RT-PCR quantitation was determined by the ΔCt method [30] for all other genes. All PCR reactions were normalised against the reference housekeeping gene glyceraldehyde-3-phosphate dehydrogenase (GAPDH).

**Table 1:** Primer sequences used in this study for qRT-PCR.

Name	Primers Sequence		Accession No.
DENV-2 capsid region	*Forward*	GCAGATCTCTGATGAATAACCAAC	D00346.1
	*Reverse*	TTGTCAGCTGTTGTACAGTCG	
GAPDH	*Forward*	GACGGCCGCATCTTCTTGTGC	NM_008084.3
	*Reverse*	TGCCACTGCAAATGGCAGCC	
SK1	*Forward*	TGTCACCCATGAACCTGCTGTCCCTGCACA	NM_001172475.1
	*Reverse*	GCCCTTCTGCACCAGTGTA	
SK2	*Forward*	TCTGGAGACGGGCTGCTTTA	NM_001172561.1
	*Reverse*	GCACCCAGTGTGAATCGAGC	
IFN-β	*Forward*	AGAAAGGACGAACATTCGGAAA	NM_010510.1
	*Reverse*	CCGTCATCTCCATAGGGATCTT	
Viperin	*Forward*	ACTCTGTCATTAATCGCTTCAACGT	NM_021384.4
	*Reverse*	TCAATTAGGAGGCACTGGAAAAC	
Ifi27l2a	*Forward*	CTGTTTGGCTCTGCCATAGGAG	NM_029803.3
	*Reverse*	CCTAGGATGGCATTTGTTGATGTGG	
IRF7	*Forward*	CACCCCCATCTTCGACTTCA	NM_001252601.1
	*Reverse*	CCAAAACCCAGGTAGATGGTGTA	
CXCL10	*Forward*	GCCGTCATTTTCTGCCTCAT	NM_021274.2
	*Reverse*	GGCCCGTCATCGATATGG	
CD4	*Forward*	CCCAGGTCTCGCTTCAGTTTG	NM_013488.2
	*Reverse*	AGGTAGGTCCCATCACCTCACAG	
CD8-β	*Forward*	GCTGGTAGTCTGCATCCTGCTTC	NM_009858.2
	*Reverse*	TTGCTAGCAGGCTATCAGTGTTGTG	

### Measurement of sphingosine kinase activity

Frozen brain tissues were homogenised and sonicated in a buffer consisting of 50 mM Tris/HCl (pH 7.4) containing 150 mM NaCl, 2 mM Na_3_VO_4_, 10 mM NaF, 10% (w/v) glycerol, 10 mM β-glycerophosphate, 0.05% (w/v) Triton X-100, 1 mM EDTA and protease inhibitors (Roche, Complete mini). SK1 and SK2 activities were selectively measured by ^32^P transfer from [γ^32^P] ATP to D-*erythro*-sphingosine under conditions of Triton X-100 for SK1 activity and 1 M KCl for SK2 activity, as previously described [31]. The SK activities were normalised against total protein content, as determined by Bio-Rad protein assay.

### S1P quantitation

S1P levels were measured as previously described [32] with some modifications. Briefly, 20 μl of brain homogenate was suspended in 200 μl methanol with 0.25% conc. HCl. Alkaline extraction of the lipids was performed by addition of 400 μl chloroform, 30 μl of 10 M NaOH, 580 μl of 2 M KCl and 200 μl methanol with 200 pmol C17-S1P and centrifuged (5 min, 16000xg). The upper aqueous/methanol phase was collected and acid extraction was performed with 40 μl conc. HCl and 300 μl chloroform. The upper phase was aspirated and 200 μl of lower choloroform phase was collected and chloroform was evaporated using a speed vacuum system. The dried lipids were resuspended in 275 μl methanol/70 mM K_2_HPO_4_ (9:1) with 1 mM EDTA by sonication in a bath sonicator for 30 sec. A derivatization mixture of 10 mg *o*-phthalaldehyde, 200 μl ethanol, 10 μl β-mercaptoethanol and 10 ml of a 3% boric acid solution (adjusted to pH 10.5 with KOH) was prepared and 25 μl of this was added to the lipid samples and incubated for 15 min at room temperature followed by centrifugation. S1P levels were then determined by HPLC analysis with an EVO C18 column (Phenomenex, Lane Cove, NSW, Australia) as previously described [32].

### Histological analysis

Mouse brains were harvested and fixed in 10% (v/v) buffered formalin. Brain tissues were embedded and block mounted in paraffin. Sections were cut into 5μm thickness, stained with haematoxylin and eosin (H&E) and examined under brightfield microscopy (BX50, Olympus).

### Statistical analysis

Statistical analyses were carried out using GraphPad Prism software version 6.07. A statistically significant difference between samples was assessed using Students unpaired *t*-test with Welch’s correction. Kaplan-Meier survival curves were analysed by log rank test (Mantel-Cox). To examine the body weight loss and appearance of clinical signs of disease Fisher’s exact test was used.

### Results

#### DENV-2 -infection induces neurological symptoms and weight loss in WT and SK1^-/-^ mice

**Fig 1:**
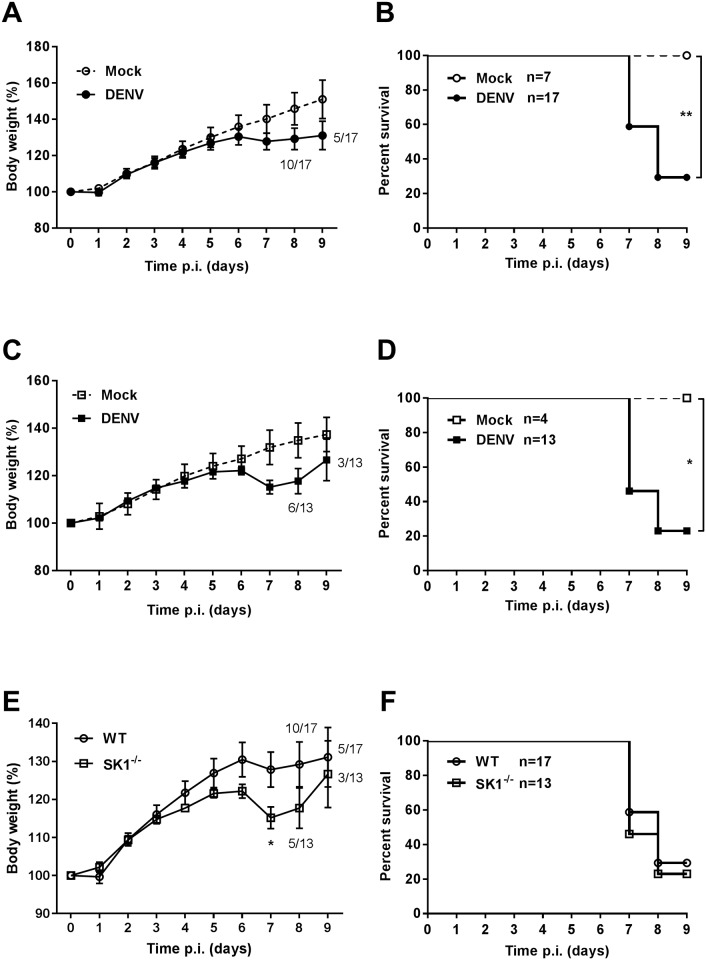
Susceptibility of WT and SK1^-/-^ mice to DENV-2 infection. 3–4 week old C57BL/6 WT (n = 17) and SK1^-/-^ (n = 13) mice were ic injected with 800 pfu DENV-2 MON601. Mock WT (n = 7) and SK1^-/-^ (n = 4) control mice were ic injected with vehicle only. Body weight and neurological symptoms were recorded. Body weight is expressed as a percentage of initial body weight. Survival reflects mice that do not show neurological symptoms or >10% of body weight loss. **A and B.** Comparison of body weight and survival curves of mock with DENV-infected WT mice; **C and D.** Comparison of body weight and survival curve of mock with DENV-infected SK1^-/-^ mice; **E and F.** Comparison of body weight percentage and survival curves of DENV-infected WT and SK1^-/-^ mice. Data are expressed as mean ± SEM. Statistical analysis of survival curves were determined by long-rank test. * = p < 0.05, ** = p < 0.005.

To define the susceptibility of WT and SK1^-/-^ mice to DENV-2 ic infection, we compared the growth rates and survival of mock and DENV-infected WT and SK1^-/-^ mice. Animals were challenged by ic injection with 800 pfu/mouse DENV-2, and body weight and neurological symptoms (slow movement, hunched posture, and reduction in hind limb movement) recorded daily. Mock-infected mice did not demonstrate any loss in body weight or neurological symptoms (Fig 1A–1D). Although there was a tendency towards a greater and earlier body weight loss in DENV-infected SK1^-/-^ mice compared with DENV-infected WT mice, there was no significant difference in terms of the overall number of mice that lost body weight, the time of onset of body weight loss, nor the percentage of mice that lost more than 7% of body weight (Table 2). When average body weight gain as a percentage of the initial body weight was analysed, results showed that both DENV-infected WT mice and SK1^-/-^ mice started to lose body weight at 6 days post infection (dpi) in comparison to mock-infected mice (Fig 1A and 1C). As expected, analysis of survival demonstrated a significantly higher mortality in DENV-infected compared to mock-infected mice (Fig 1B and 1D). Comparison of body weight loss and survival of DENV-infected WT and SK1^-/-^ mice support the trends in Table 2 and demonstrate that DENV-infected SK1^-/-^ mice show a significantly greater weight loss at 7 dpi than WT mice (Fig 1E). At this time point, 7 out of 17 WT mice (~41%) and 7 out of 13 SK1^-/-^ mice (~54%) were sacrificed due to either excessive body weight loss and/or appearance of signs of DENV-induced neurovirulence. This is reflected by an apparent higher survival rate of WT compared to SK1^-/-^ mice at 7 and 8 dpi, although this was not statistically significant (Fig 1F). These results suggest that C57BL/6 mice deficient in SK1 tend to be more susceptible to body weight loss following DENV-2 infection than their counterpart WT mice but the overall disease profile is comparable.

**Table 2:** Summary of body weight loss and signs of DENV-induced neurovirulence in WT and SK1^-/-^ mice and analysed by Fisher’s exact test.

Criteria	Mice strains				*P*
	WT		SK1^-/-^		
	Number	%	Number	%	
**Body weight loss**					
Weight loss	15	88.24	12	92.31	0.999
No weight loss	2	11.76	1	7.69	
**% Body weight loss**					
< 7%	8	53.33	2	16.67	0.107
> 7%	7	46.67	10	83.33	
**Days pi onset loss**					
< 7 dpi	3	20.00	5	41.67	0.398
≥ 7 dpi	12	80.00	7	58.33	
**Appearance of neurovirulence**					
Neurovirulence	10	58.82	9	69.23	0.708
No neurovirulence	7	41.18	4	30.77	

#### DENV-2 RNA levels are not significantly different between WT and SK1^-/-^ mice but correlate with DENV-induced disease

**Fig 2:**
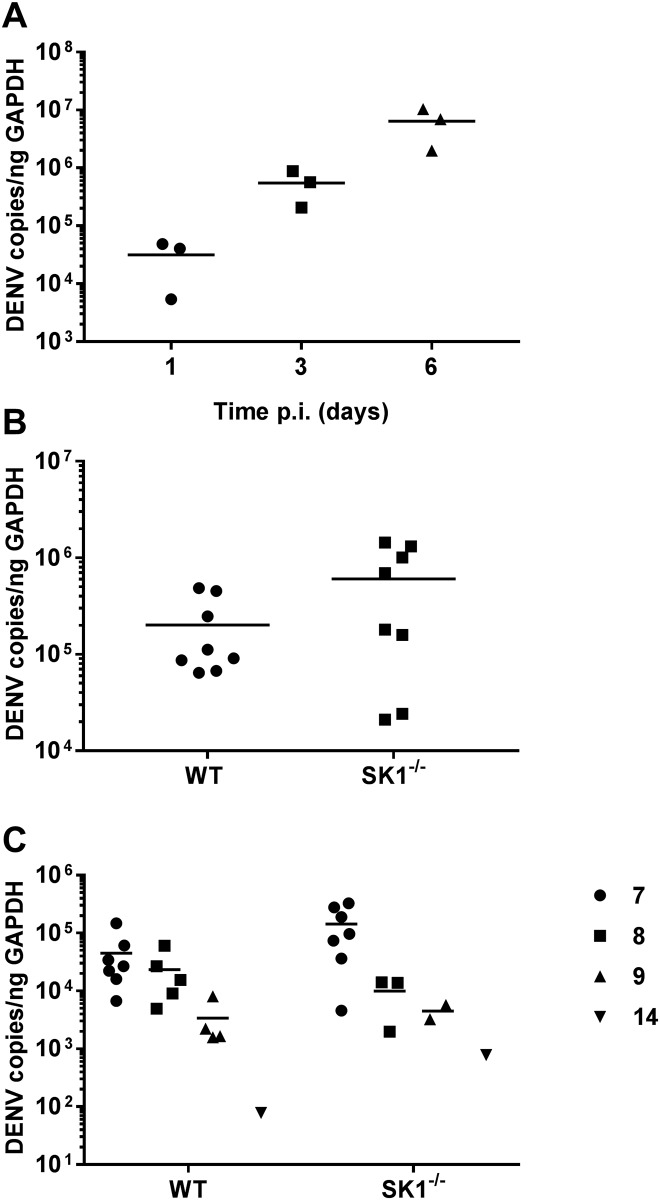
DENV-2 RNA levels increase WT and SK1-/- mice following ic DENV infection. WT and SK1^-/-^ mice were ic infected with DENV, as in Fig 1. RNA was isolated from infected mice brain tissues and analysed by real time qRT-PCR for DENV. A. Total DENV-2 RNA increases with time in WT mice, n = 3 at each time point; B. RNA was isolated from infected WT and SK1^-/-^ mice brain tissues at 3 dpi, n = 8 for each strain; C. RNA was isolated from infected mice brain tissues at the time of humane sacrifice, representing 7 (n = 7), 8 (n = 5), 9 (n = 4) or 14 (n = 1) dpi for WT and 7 (n = 7), 8 (n = 3), 9 (n = 2) or 14 (n = 1) dpi for SK1^-/-^ mice. Each symbol represents an individual mouse sample. Data represent average PCR values from individual mice. Statistical significance was assessed by unpaired Student *t*-test.

DENV replication in the brain following ic injection was validated by analysis of the time course of DENV RNA changes in WT mice. qRT-PCR analysis demonstrated increasing DENV RNA level with time with 3 dpi representing an early time point where DENV RNA levels were increasing (Fig 2A). Eight WT or SK1^-/-^ mice were injected ic with DENV-2 and brain tissues harvested at 3 dpi. DENV RNA levels at 3 dpi tended to be higher in SK1^-/-^ compared with WT mice (p = 0.083), but were not significantly different (Fig 2B). RNA levels were also quantitated in WT and SK1^-/-^ mice at the termination of experiments. DENV RNA level was significantly higher at day 7–8, than at 9–14 dpi in both WT and SK1^-/-^ mice (Fig 2C). The sacrifice of mice at 7–8 dpi was due to symptomatic presentation and ethical termination, but those sacrificed at 9–14 dpi showed body weight loss but lacked neurological symptoms and represented an elective time for termination of the experiment. Based on the disease presentation and comparable level of DENV RNA at 7 and 8 dpi, we have considered this combined time point as ‘end-stage disease’. We have indicated the 9–14 dpi animals in Figs 4–7 as half-filled symbols but excluded these animals from our statistical analysis since they did not show DENV-induced disease. It should be noted, however that the inclusion of the 9–14 dpi mice in the data set did not influence the statistical outcomes. At end-stage disease (7–8 dpi), DENV RNA levels again tended to be higher in SK1^-/-^ mice brains in comparison to WT mice (Fig 2C) but were not significantly different (p = 0.116). These data suggest that DENV-2 can replicate and cause body weight loss and neurological symptoms when introduced directly into the mouse brain, with a moderate but not statistically significant increase in DENV RNA in SK1^-/-^ compared to WT mice.

#### DENV-2 infection induces IFN-β and interferon-stimulated genes (ISGs) but this is not different in WT and SK1^-/-^ mice

**Fig 3:**
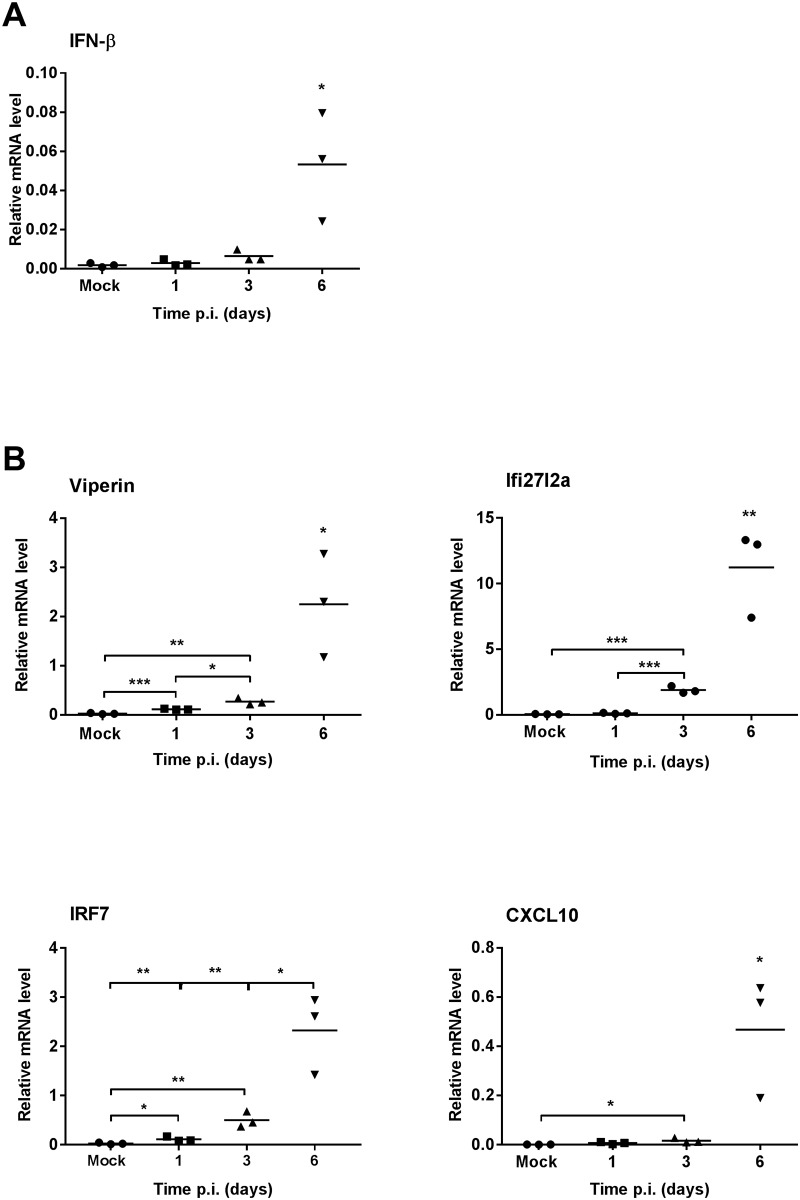
The time course of induction of IFN-β and ISGs in WT mice following ic infection with DENV-2. WT mice (n = 3 at each time point) were ic infected with DENV, as in Fig 1. At the time point indicated RNA was isolated from infected mice brain tissues and analysed by real time qRT-PCR for **A.** IFN-β; **B.** ISGs viperin, Ifi27l2a, IRF7 and CXCL10. Data represent average PCR values from individual mice and normalized against GAPDH by ΔCt method. Statistical significance was assessed by unpaired student *t*-test * = p < 0.05, ** = p < 0.005, *** = p < 0.0005.

Type I interferon, such as IFN-β, is an important part of the early immune response to DENV infection and to define this response following DENV-2 infection in the brain we analysed mRNA levels for IFN-β and the ISG’s viperin, Ifi27l2a, IRF7 and CXCL10 in WT mice by qRT-PCR. Results show increased IFN-β mRNA, late in the course of infection at 6 dpi (Fig 3A). In comparison to this late induction of IFN-β, all the ISGs analysed were rapidly and significantly induced, as early as 1 dpi, for viperin and IRF7 or 3 dpi, for Ifi27l2a and CXCL10 (Fig 3B).

**Fig 4:**
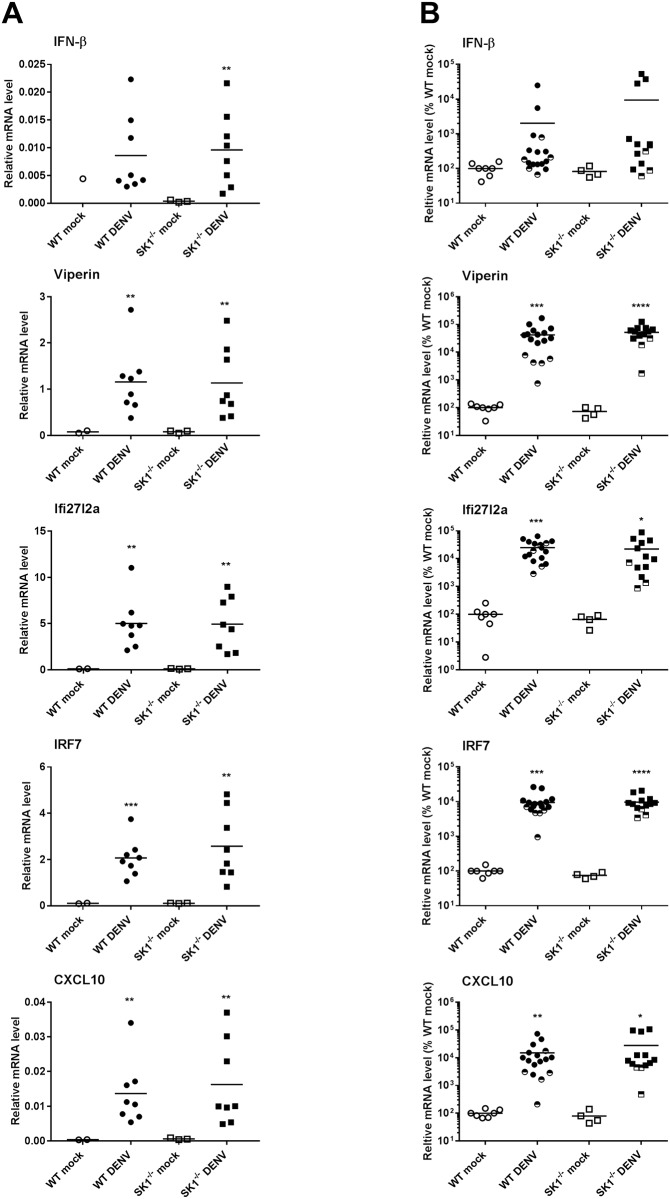
Induction of IFN-β and ISGs in WT and SK1^-/-^ mice following ic infection with DENV-2. WT and SK1^-/-^ mice were ic infected with DENV, as in Fig 1. RNA was isolated from infected mice brain tissues and analysed by real time qRT-PCR **A.** at 3 dpi for IFN-β, viperin, Ifi27l2a, and CXCL10. n = 8 WT and SK1^-/-^ DENV-infected, n = 2 WT mock and n = 3 SK1^-/-^ mock-infected mice; **B.** at end stage disease for IFN-β, viperin, Ifi27l2a, and CXCL10. n = 12 WT and n = 10 SK1^-/-^ DENV-infected, n = 7 WT mock and n = 4 SK1^-/-^ mock-infected mice. Data points representing non-symptomatic animals (9–14 dpi) are indicated by the half-filled symbols. Statistical analysis has been performed on symptomatic DENV-infected mice only (7–8 dpi), excluding n = 5 WT and n = 3 SK1^-/-^ at 9/14 dpi. Data represent average PCR values from individual mice and normalized against GAPDH by ΔCt method. Statistical significance was assessed by unpaired student *t*-test. * = p < 0.05, ** = p < 0.005, *** = p < 0.0005, **** = p < 0.00005.

Comparison of responses in WT and SK1^-/-^ mice at 3 dpi, showed that IFN-β and ISGs (viperin, Ifi27l2a, IRF7 and CXCL10) mRNA levels were induced following DENV-infection but did not differ between WT and SK1^-/-^ mice (Fig 4A). At end stage disease, IFN-β mRNA levels tended to be increased (p = 0.224 WT; 0.086 SK1^-/-^), while the ISGs viperin, Ifi27l2a, IRF7 and CXCL10 were all significantly and highly induced by DENV-infection (Fig 4B). As seen at 3 dpi (Fig 4A), IFN-β and ISG mRNA levels were not significantly different between DENV-infected WT and SK1^-/-^ mice at end stage disease (Fig 4B). These data suggest that ISGs are induced early during DENV infection in the mouse brain, prior to the detectable induction of IFN-β, with the induction of ISGs persisting until end stage disease. The lack of SK1 however, has no effect on this response.

#### DENV-2 infection in the brain does not alter the SK/S1P axis

**Fig 5:**
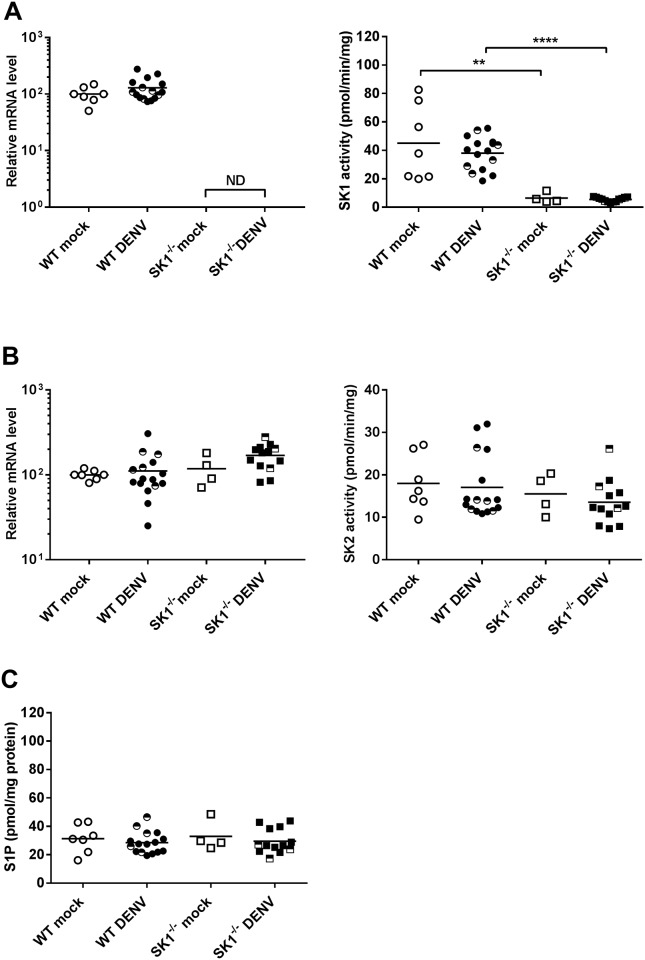
Definition of the SK/S1P axis in WT and SK1^-/-^ mice following ic infection with DENV-2. WT and SK1^-/-^ mice were ic infected with DENV, as in Fig 1 and at end stage disease, brain tissue was harvested and snap frozen or stored in TRIzol. RNA was extracted from TRIzol and lysates prepared in EB buffer from snap frozen tissue. **A.** SK1 mRNA was determined by qRT-PCR (left panel) and isoenzyme specific SK1 activity assay (right panel); **B.** SK2 mRNA was determined by qRT-PCR (left panel) and isoenzyme specific SK2 activity assay (right panel); **C.** S1P was quantitated in brain lysates by HPLC. n = 11 WT and n = 10 SK1^-/-^ DENV-infected, n = 7 WT mock and n = 4 SK1^-/-^ mock-infected mice. PCR data represent average PCR values from individual mice and normalized against GAPDH by ΔCt method. SK activity data and S1P quantitation are expressed relative to total protein quantitation. Statistical significance was assessed by unpaired student *t*-test. ** = p < 0.05, **** = p < 0.00005. ND, not detected. Data points representing non-symptomatic animals (9–14 dpi) are indicated by the half-filled symbols. Statistical analysis has been performed on symptomatic DENV-infected mice only, excluding n = 5 WT and n = 3 SK1^-/-^ at 9/14 dpi.

To validate the lack of SK1 in SK1^-/-^ mice and assess the impact of this and DENV-infection on the SK/S1P axis in the brain, we quantitated SK1 and SK2 mRNA, performed isoform-specific SK activity assays and determined S1P levels in the brain at end stage disease. qRT-PCR for SK1 mRNA and *in vitro* assays for SK1 activity in the brain verified the lack of SK1 in the mice (Fig 5A). Additionally, neither SK1 mRNA nor activity were altered in DENV-infected WT mice (Fig 5A). The lack of SK1 could be compensated for by an increase in SK2 levels. Results, however demonstrated no change in SK2 mRNA nor SK2 activity in SK1^-/-^ mice brains (Fig 5B). Again, neither SK2 mRNA or SK2 activity was altered in DENV-infected WT or SK1^-/-^ mice (Fig 5B). Further, S1P levels were quantitated in brain lysates. Notably, S1P levels were not different between WT and SK1^-/-^ mice, nor did they change following DENV-infection of either mouse strain (Fig 5C).

#### DENV-2 infection induces CD8^+^ but not CD4^+^ T-cell infiltration in the brain

**Fig 6:**
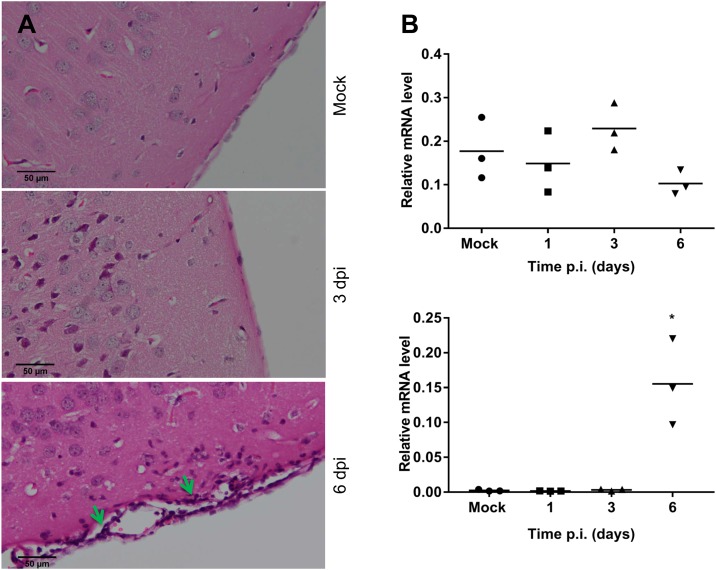
T-cell infiltration in the brain of WT mice following ic infection with DENV-2. WT mice were ic infected with DENV, as in Fig 1 and brain tissue was harvested. **A.** at 3 and 6 dpi, tissue was fixed and processed for H&E staining. Images are representative of n = 3 mice. Arrows indicate sites of cellular infiltrate; **B.** at the indicated time point RNA was extracted and CD4 and CD8 mRNA determined by qRT-PCR with n = 3 mice at each time point. Data represent average PCR values from individual mice and normalized against GAPDH by ΔCt method. Statistical significance was assessed by unpaired student *t*-test. * = p < 0.05.

CXCL10 which we have demonstrated to be induced (Figs 3 and 4), and S1P which is unchanged following DENV-infection, can both influence T-cell migration. Thus, we examined cell infiltration by histological H&E staining and the presence of mRNA for the T-lymphocyte markers, CD4 and CD8 by qRT-PCR in WT mouse brain tissue throughout the course of DENV ic infection. H&E staining of fixed brain tissue demonstrated the presence of a cellular infiltrate at 6 dpi in DENV compared to mock-infected mouse brain (Fig 6A). Results show no change in the level of CD4, but a marked and significant increase in CD8 by day 6 pi (Fig 6B), suggesting CD8+ T-cell infiltration following DENV-infection in the WT mouse brain.

**Fig 7:**
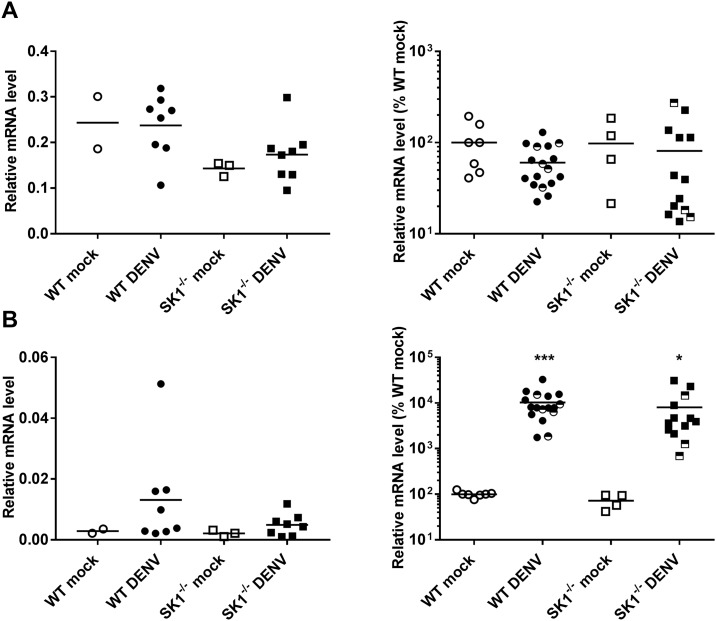
T-cell infiltration in the brain of WT and SK1^-/-^ mice following ic infection with DENV-2. WT and SK1^-/-^ mice were ic infected with DENV, as in Fig 1 and brain tissue harvested at 3 dpi and end stage disease. RNA was isolated from infected mice brain tissues and analysed by real time qRT-PCR for **A.** CD4 mRNA; **B.** CD8 mRNA. At 3 dpi n = 8 WT and SK1^-/-^ DENV-infected, n = 2 WT mock and n = 3 SK1^-/-^ mock-infected mice. At end stage disease (7–8 dpi) n = 12 WT and n = 10 SK1^-/-^ DENV-infected, n = 7 WT mock and n = 4 SK1^-/-^ mock-infected mice. Data points representing non-symptomatic animals (9–14 dpi) are indicated by the half-filled symbols. Statistical analysis has been performed on symptomatic DENV-infected mice only, excluding n = 5 WT and n = 3 SK1^-/-^ at 9/14 dpi. Data represent average PCR values from individual mice and normalized against GAPDH by ΔCt method. Statistical significance was assessed by unpaired student *t*-test. * = p < 0.05, ** = p < 0.005, *** = p < 0.0005.

The levels of CD4 and CD8 mRNA were compared by qRT-PCR in WT and SK1^-/-^ mice at 3 dpi and end stage disease (Fig 7). Results again demonstrate a lack of increase in CD4 mRNA levels following DENV-infection of WT or SK1^-/-^ mice at either time point pi and no significant difference between DENV-infected WT and SK1^-/-^ mice (Fig 7A). The levels of CD8 mRNA, however were significantly higher in DENV-infected WT and SK1^-/-^ mice compared to their mock-infected controls at end stage disease (Fig 7B), but once again there was no significant difference in CD8 mRNA between DENV-infected WT and SK1^-/-^ mice. These data suggest that CD8+ but not CD4+ T-lymphocytes infiltrate the brain of DENV-infected mice but there is no major role of SK1 in driving CD8+ T-cell migration into the brain during DENV-infection.

### Discussion

A growing number of studies have reported SK1 as a factor that contributes to the modulation of several viral infections *in vitro* [10]. Our own studies have suggested changes in SK1 in response to DENV-2 infection [15, 33] and a role for SK1 early in infection in promoting DENV-induction of ISGs *in vitro* [17]. In the study here we have investigated the role of SK1 during DENV-2 infection *in vivo* using immunocompetent C57BL/6 mice and direct ic DENV infection.

Following DENV-2 infection, as expected, WT and SK1^-/-^ mice exhibited body weight loss and signs of neurovirulence compared to mock-infected mice. This reflects the pathogenicity and virulence of the DENV-2 MON 601 strain in mouse brain, as has been previously reported [29, 34]. Similar to other studies of DENV ic infection, not all mice develop neurological symptoms [29, 34]. We have further shown here that symptomatic DENV-infection with body weight loss and neurological signs is positively associated with the level of DENV RNA in the brain with a lower level of DENV RNA in the brains of asymptomatic mice at 9–14 dpi. The data from these mice has been represented graphically but we have excluded these from our statistical analysis. Interestingly, these asymptomatic mice strikingly group together with lower induction of viperin compared to symptomatic mice (Fig 4B) but are interspersed with the symptomatic mice in terms of responses such as CD8+ mRNA levels (Fig 7B). This suggests an association of viperin with symptomatic DENV-infection in the brain, and this remains to be investigated further.

Mice that are deficient in SK1 showed moderately greater and earlier body weight loss and tended to have higher levels of DENV RNA following DENV-2 infection than their counterpart WT mice. Since SK1 is a pro-survival factor [35], it is possible that without SK1 mice are more prone to weight loss and growth deficiencies. Additionally, the trend towards an increase in DENV RNA in SK1^-/-^ mice, although not statistically significant (p = 0.116), is consistent with our recent data showing enhancement of DENV-2 infection in SK1^-/-^ primary mouse embryonic fibroblast (MEF), that we demonstrated is associated with a reduced ability of DENV to induce ISGs in the absence of SK1 [17]. Thus, we assessed the SK1-dependency of the induction of ISGs in this DENV-brain infection model. We chose to analyse the mRNA level of selected important type I IFN driven ISGs: viperin [36], IRF7 [37], and CXCL10 [38] that have been described previously as antiviral factors against DENV and we have previously shown to be induced in a SK1-dependent manner in response to DENV infection *in vitro* [17]. Ifi27l2a was also assessed as an ISG, previously reported to be induced in the brain in response to West Nile Virus (WNV) infection [39], and for which the response to DENV-infection has not been previously defined. At 6 dpi and end stage disease the level of IFN-β mRNA tended to be increased. In contrast there was a more rapid (1–3 dpi) and significant induction of ISGs viperin, Ifi27l2a, IRF7 and CXCL10 that persisted until end stage disease. IFIT1 and OAS1, which are similarly anti-viral against DENV [40, 41] were also induced rapidly but were not analysed in our complete experimental set (data not shown). This is the first report of induction of these ISGs in the brain following DENV-infection and the first report of DENV-induction of Ifi27l2a. The discordance between the induction of IFN-β and these ISGs contrasts to studies that show IFN-β to be a major driver of neuronal ISGs response during WNV infection in the brain [42, 43] and ISGs following DENV-infection in some cells [16, 44]. This suggests that IFN-β may not be the major driver of induction of these ISGs following DENV infection in the brain. Potentially, other factors such as IFN-γ or λ may be more important in this tissue [45–47]. Studies have shown the activation of microglial cells, an increase in neurological disease severity when these cells are depleted and a role for microglia in production of cytokines and chemokines such as RANTES, IFN-γ, IL-6, MCP-1 and MCP5 following DENV-infection in the brain [48]. Similarly, in our model of DENV-ic infection, these resident microglial cells may be the source of the induction of ISGs we have observed here.

The induction of ISGs is evident prior to the presence of symptomatic infection but was not significantly different between WT and SK1^-/-^ mice. This contrasts to our prior data from *in vitro* studies where embryonic fibroblasts from SK1^-/-^ mice or cells treated with an SK1 inhibitor (SK1-I) show reduced DENV-induction of ISGs such as viperin, IFIT1 and CXCL10 [17]. Similarly, genetic deletion or chemical inhibition of SK1 reduced the IL-1 induced expression of CXCL10 [6]. This suggests that in contrast to MEF and peripheral responses, the deficiency of SK1 does not compromise the induction of ISGs in the model herein of DENV-infection of the brain. Further, that lack of a major effect of the lack of SK1 on DENV-disease suggests that similarly SK1-dependent inflammatory pathways are not involved in regulating DENV replication and pathogenicity in the brain. This contrasts to LPS [49] or ischemia-induced neuroinflammation [50] where SK1 has been shown to be present in microglia and to promote the induction of factors such as TNF-α, IL-1 and nitric oxide [49] but intriguingly a lack of SK1 exacerbates LPS induced neuroinflammation [51]. Our observed lack of influence of SK1 on DENV in the brain may be due to pathogen or stimulus specific roles for SK1 in brain inflammation or tissue specific roles for SK1, where in the brain SK2 is known to be the dominant SK isoform [52]. The brain is an immune privileged site and previous studies have also reported differences in the expression of ISGs between the brain and other tissues. For example, mRNA levels for genes such as OAS, MDA5, and STAT1 as well as ISGs IFIT1, IFIT2, and ISG15 are greater in the liver than the brain of mice [53, 54]. Studies also showed expression of ISGs vary between the different cell types within the brain itself. For example, the basal and inducible levels of OAS were lower in neurons than microglia in response to MHV infection [53] and higher levels of both basal and induced ISGs (e.g. IFIT1 and IRF7) are observed in microglia than oligodendrocytes [53, 55]. Additionally, mouse cortical neurons fail to express basal levels of ISG54 and ISG56 [56] while distinct neuronal subsets in the brain differentially express Ifi27, IRF1 and viperin that make these cells more susceptible to WNV infection [43]. Thus, although we saw no overall effect of a lack of SK1 in a whole brain extract, and in situ analysis of SK1 and ISGs in different cells of the brain may be informative.

The SK/S1P axis is a finely regulated system [57]. Our findings demonstrated that the lack of SK1 in the mouse brain was not compensated for by changes in SK2 mRNA or activity, consistent with prior studies in the periphery [28, 58]. Further, SK1^-/-^ mice do not have altered levels of S1P in the brain. Additionally, DENV-infection did not affect SK1 or SK2 mRNA or activity in the brain. This contrasts to the early increase in SK1 activity that we have documented in DENV-infected EC [16] and inhibition late in infection in a number of cell types *in vitro* [15], which again may reflect tissue-specific responses of SK1 to infection. Further, SK2 mRNA or activity or S1P levels in the brain were not different following DENV-infection of WT and SK1^-/-^ mice.

Following viral infection, one would expect to see an immune cell infiltrate to the site of infection. We demonstrated a marked cell infiltrate in DENV-infected mouse brain by H&E staining and further characterised the nature of this cellular infiltrate as CD8+ but not CD4+ T-cells by RT-PCR analysis, although we did not analyse the presence of monocytes, a cell type also known to infiltrate the brain in WNV infection [59, 60]. A prior study has shown both CD4+ and CD8+ T cells infiltration into the brain following DENV-3 challenge in C57BL/6 mice [25], or DENV-2 challenge in BALB/c mice [34]. Further, Hsieh *et al*. showed predominant CD8+ compared to CD4+ T cell infiltration in DENV-infected mouse brain [38]. Similarly, the infiltration of T cells has been demonstrated in mouse brains during infection with other flaviviruses. Increased CD8+ but not CD4+ mRNA levels were detected in Japanese encephalitis virus (JEV)-infected mouse brains [61], and similarly CD8+ T-cells infiltrate WNV-infected mouse brains [62] and following ic challenge with the Alphavirus, Semliki Forest virus (SFV) [63]. In contrast, both CD4+ and CD8+ T cells have been reported in mice challenged with a WNV [60, 64] and Yellow fever (YF)-17D virus [65]. Thus, there is an overall consensus from a number of *flavivirus* and other virus infections in the brain that CD8+ T-cells are a major infiltrating cell type, and our data is consistent with this suggestion.

CXCL10 has been implicated in T-cell migration into the brain in WNV and SFV infections [63, 66] and S1P is an important regulator of T-cell migration from lymph nodes in the periphery [67]. CXCL10 was induced following DENV-brain infection of WT mice in our study but S1P was unchanged, supporting a potential role of CXCL10 but not S1P in driving CD8+ T-cell infiltration during DENV-infection of the brain. The induction of CXCL10 mRNA at 3 dpi and prior to the onset of T-cell infiltration at 6 dpi further supports this association. Additionally, in our study a lack of SK1 did not affect DENV-induced responses of CXCL10 or S1P and consistent with this, a lack of SK1 did not affect T-cell infiltration in the brain during DENV infection.

In summary, in this study we have shown that SK1 has a moderate effect on body weight loss and DENV RNA levels following DENV-2 infection in the mouse brain but has no overall major impact on DENV-induced disease. While DENV-infection induced ISGs and CD8+T-cell infiltration, the SK/S1P axis is not affected by DENV-infection and none of these factors were significantly affected by the absence of SK1. This data has defined innate responses to DENV-infection in the brain and demonstrate that in contrast to our studies showing a role of SK1 in promoting ISG induction following DENV-infection *in vitro*, and other studies demonstrating a role for SK1 in non-infectious neuroinflammation, in the scenario of DENV-infection of the brain, SK1 does not play a role in these processes.
